# High frequency atomic tunneling yields ultralow and glass-like thermal conductivity in chalcogenide single crystals

**DOI:** 10.1038/s41467-020-19872-w

**Published:** 2020-11-27

**Authors:** Bo Sun, Shanyuan Niu, Raphael P. Hermann, Jaeyun Moon, Nina Shulumba, Katharine Page, Boyang Zhao, Arashdeep S. Thind, Krishnamurthy Mahalingam, JoAnna Milam-Guerrero, Ralf Haiges, Matthew Mecklenburg, Brent C. Melot, Young-Dahl Jho, Brandon M. Howe, Rohan Mishra, Ahmet Alatas, Barry Winn, Michael E. Manley, Jayakanth Ravichandran, Austin J. Minnich

**Affiliations:** 1 Division of Engineering and Applied Science, California Institute of Technology, Pasadena, CA 91125 USA; 2 Tsinghua-Berkeley Shenzhen Institute and Tsinghua Shenzhen International Graduate School, Guangdong Provincial Key Laboratory of Thermal Management Engineering and Materials, Tsinghua University, 518055 Shenzhen, Guangdong China; 3 Mork Family Department of Chemical Engineering and Materials Science, University of Southern California, Los Angeles, CA 90089 USA; 4 School of Earth, Energy and Environmental Sciences, Stanford University, Stanford, CA 94305 USA; 5 Materials Science and Technology Division, Oak Ridge National Laboratory, Oak Ridge, TN 37831 USA; 6 Neutron Scattering Division, Oak Ridge National Laboratory, Oak Ridge, TN 37831 USA; 7 Institute of Materials Science and Engineering, Washington University in St. Louis, St. Louis, MO 63130 USA; 8 Materials and Manufacturing Directorate, Air Force Research Laboratory, Wright-Patterson AFB, Dayton, OH USA; 9 Department of Chemistry, University of Southern California, Los Angeles, CA 90089 USA; 10 Loker Hydrocarbon Research Institute, University of Southern California, Los Angeles, CA 90089 USA; 11 Core Center of Excellence in Nano Imaging, University of Southern California, Los Angeles, CA 90089 USA; 12 School of Electrical Engineering and Computer Science, Gwangju Institute of Science and Technology, Gwangju, 61005 South Korea; 13 Department of Mechanical Engineering and Materials Science, Washington University in St. Louis, St. Louis, MO 63130 USA; 14 Advanced Photon Source, Argonne National Laboratory, Argonne, IL 60439 USA; 15 Ming Hsieh Department of Electrical Engineering, University of Southern California, Los Angeles, CA 90089 USA

**Keywords:** Structure of solids and liquids, Materials for energy and catalysis, Quantum mechanics

## Abstract

Crystalline solids exhibiting glass-like thermal conductivity have attracted substantial attention both for fundamental interest and applications such as thermoelectrics. In most crystals, the competition of phonon scattering by anharmonic interactions and crystalline imperfections leads to a non-monotonic trend of thermal conductivity with temperature. Defect-free crystals that exhibit the glassy trend of low thermal conductivity with a monotonic increase with temperature are desirable because they are intrinsically thermally insulating while retaining useful properties of perfect crystals. However, this behavior is rare, and its microscopic origin remains unclear. Here, we report the observation of ultralow and glass-like thermal conductivity in a hexagonal perovskite chalcogenide single crystal, BaTiS_3_, despite its highly symmetric and simple primitive cell. Elastic and inelastic scattering measurements reveal the quantum mechanical origin of this unusual trend. A two-level atomic tunneling system exists in a shallow double-well potential of the Ti atom and is of sufficiently high frequency to scatter heat-carrying phonons up to room temperature. While atomic tunneling has been invoked to explain the low-temperature thermal conductivity of solids for decades, our study establishes the presence of sub-THz frequency tunneling systems even in high-quality, electrically insulating single crystals, leading to anomalous transport properties well above cryogenic temperatures.

## Abstract (web summary)

Defect-free crystals showing the glassy trend of low thermal conductivity with a monotonic increase with temperature are desirable but rare. Here, the authors observe atomic tunneling associated with low and glass-like thermal conductivity in a hexagonal perovskite chalcogenide single-crystal BaTiS_3_.

## Introduction

Low thermal conductivity crystals have been extensively researched for applications such as thermoelectrics^1–6^ and are typically realized by introducing crystalline defects that scatter phonons^7–9^. This strategy has been demonstrated in diverse classes of materials including multilayers^10,11^, polycrystalline semiconductors^12,13^, and solids containing nanoprecipitates^14,15^, among many others. Crystalline materials that exhibit intrinsically low thermal conductivity typically possess large anharmonic force constants, as in SnSe^16^ and Tl_3_VSe_4_^4^; a complex crystal structure leading to a large phonon scattering phase space^7,17^; or a naturally occurring inhomogeneous nanoscale structure, as in AgSbTe_2_^2^, otherwise remaining free of structural defects. Such homogeneous single crystals with low defect concentrations usually exhibit the familiar trend of decreasing thermal conductivity with increasing temperature above the Debye temperature^18^. Within the perovskite material family, the thermal conductivity varies from 1 to 20 W m^−1^ K^−1^ for the simplest ABO_3_-type perovskite oxides^19–21^ and chalcogenides^22^, and <1 W m^−1^ K^−1^ for organic–inorganic hybrid perovskites^23^ and inorganic halides^24^, although all of these materials follow the crystalline trend of thermal conductivity with temperature.

Only a few compounds have been reported to deviate from this trend. While a material with a complex or low-symmetry crystal structure does not necessarily possess a glassy thermal conductivity, most materials exhibiting the trend are structurally complex. Examples include NaNbO3^21^ with a low-symmetry monoclinic or orthorhombic crystal structure at room temperature^25^, orthorhombic CsBiNb2O7 with 22 atoms per primitive cell^26^, polydiacetylene single crystals^27^, Yb-based zintls^28^, and clathrates^5,6,29^. The glassy thermal conductivity of clathrates, in particular, have been extensively investigated^30–32^. However, unambiguously identifying the origin of the glassy trend has proved challenging. For instance, while early works on clathrates hypothesized that two-level systems were responsible^29,30^, later works suggested that charge carrier scattering could play a role^33^ as the samples were electrically conducting. Further, direct detection of tunneling of heavy structural atoms is generally not possible with scattering methods as the tunneling splitting energies are expected to be sub GHz^34^, which is too low to affect thermal conduction except at temperatures less than around 1 K. As a result, the mechanism underlying the glassy thermal conductivity of single crystals remains unclear.

Here, we report measurements of the thermal conductivity of single crystals of electrically insulating hexagonal perovskite chalcogenide BaTiS_3_ with a highly symmetric and simple primitive unit cell. We find that the thermal conductivity of this material is among the lowest ever reported for single crystals, and further exhibits a glass-like temperature dependence with ultralow thermal conductivity extending down to cryogenic temperatures. Elastic and inelastic scattering measurements reveal the origin of this unusual trend as scattering from an atomic tunneling two-level system formed by the Ti atom residing in a shallow double-well potential. Our study provides the first direct observation of a two-level system of sufficiently high frequency to disrupt thermal conduction in high-quality, electrically insulating single-crystalline materials over an extended temperature range, a result that is of both fundamental and practical interest.

## Results

### Crystal structure of BaTiS_3_

**Fig. 1:**
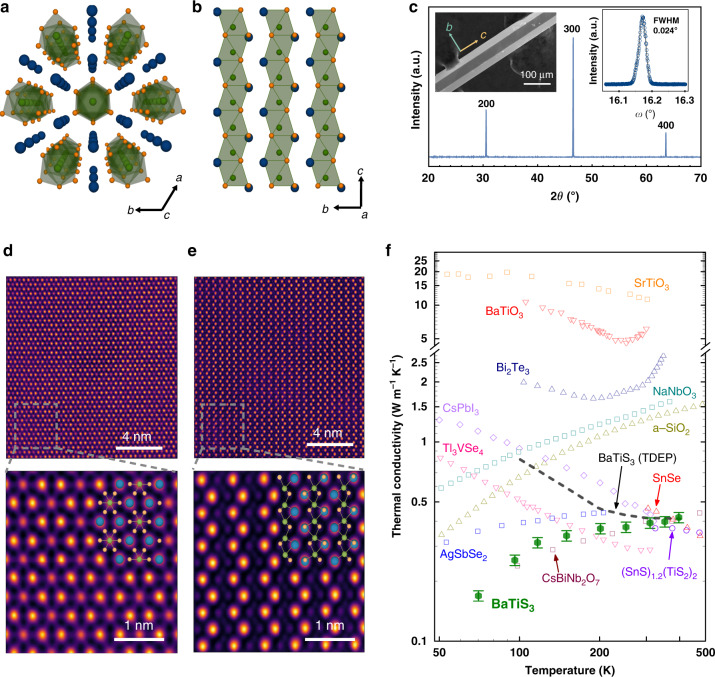
Structural and thermal transport properties of BaTiS_3_ single crystal. Illustration of the BaTiS_3_ structure in perspective view along *c* axis (**a**) and projected down *a* axis (**b**). Blue and orange spheres denote Ba and S atoms, respectively. Octahedra formed by Ti and six surrounding S atoms are highlighted in green. **c** XRD measurement of a crystal to identify the crystallographic directions. The left inset panel of **c** is an SEM image of the sample for which thermal conductivity was measured using TDTR. The right inset panel of **c** is the rocking curve for the 200 reflections. STEM HAADF images showing *c* axis (**d**) and *a* axis (**e**) projection of BaTiS_3_. The bottom panels show the high-resolution HAADF images with overlaid atomic models. **f** Measured thermal conductivity versus temperature for BaTiS_3_ (green) and other materials from literature. First-principles calculations using temperature-dependent effective potential (TDEP) method are also shown (black dashed line). Data for BaTiO_3_ and SrTiO_3_, amorphous silica (a-SiO_2_), Bi_2_Te_3_, CsBiNb_2_O_7_, CsPbI_3_, NaNbO_3_, Tl_3_VSe_4_, AgSbSe_2_, SnSe and (SnS)_1.2_(TiS_2_)_2_ are from refs. ^4,16,18,19,21,26,41,69,70^, respectively. The estimation of error bars is detailed in Supplementary Information.

We synthesized needle-like single crystals of BaTiS_3_ with dimensions up to  ~2 cm  ×  100 μm ×  50 μm using chemical vapor transport^35,36^. BaTiS_3_ has a BaNiO3-type hexagonal perovskite structure in which the octahedra of Ti coordinated by S atoms share common faces, resulting in chain-like structures along the *c* axis. These parallel chains possess strong intra-chain bonding and weak electrostatic interactions along *a*/*b*-axes, as shown in Fig. 1a, b. From the X-ray diffraction (XRD) measurement (Fig. 1c) and a rotational XRD map (see Supplementary Information Fig. S1), the crystallographic orientation of the BaTiS_3_ single crystal was determined. The termination plane was the *bc*-plane with the *c* axis as the long edge, as depicted in the scanning electron microscopy (SEM) image in the inset of Fig. 1c. The rocking curve of the 200 reflections is also shown in the inset of Fig. 1c. The sharp full-width-at-half-maximum (FWHM) of 0.024° indicates high crystalline quality. We also performed refinements of the single-crystal XRD measurements, which yielded a structure of *P*6_3_*mc*, in agreement with a previous report^37^. The refinements on multiple single-crystal samples showed that the crystals were stoichiometric within the limits of the measurement technique.

To further ascertain the quality of the BaTiS_3_ crystals, we performed atomic-scale imaging using scanning transmission electron microscopy (STEM). Figure 1d, e shows wide field-of-view high-angle annular dark-field (HAADF) images of a BaTiS_3_ crystal along with *c* and *a* axis projections, respectively. The zoomed-in view of atomic resolution HAADF images overlaid with corresponding atomic models are also shown. In this imaging mode, the intensity of an atomic column is approximately proportional to the squared atomic number (~*Z*^2^) of the elements in the column^38^. The corresponding selected area electron diffraction (SAED) patterns are available in Supplementary Information (Supplementary Fig. S2). We did not observe any line or planar defects, confirming the high quality of the BaTiS_3_ crystals. These STEM images provide a detailed view of the atomic positions compared to our past investigation^35^ and help rule out the presence of any extended defects or large concentrations of point defects.

### Glass-like and ultralow thermal conductivity

We measured the thermal conductivity of BaTiS_3_ using time-domain thermoreflectance (TDTR)^39,40^. Owing to the sample geometry and the low thermal conductivity, we can only measure the cross-plane thermal conductivity or thermal conductivity perpendicular to the *c* axis. We determined the thermal conductivity by fitting the measured TDTR signal using a standard thermal diffusion model^39^. (See Supplementary Information for details of the implementation, raw data, analysis, and uncertainty estimation of the TDTR measurements). The measured thermal conductivity of BaTiS_3_ from 70 to 400 K is shown in Fig. 1f. The measured thermal conductivity *κ* of BaTiS_3_ at room temperature is only 0.39 W m^−1^ K^−1^. This value is more than an order of magnitude smaller than that of BaTiO_3_^19^ and is comparable to the lowest thermal conductivity reported for single crystals such as Tl_3_VSe_4_^4^, CsBiNb_2_O_7_^26^, and CsPbI_3_^41^. Further, the measurements show an anomalous trend of increasing thermal conductivity with increasing temperature over the entire measured temperature range that is unexpected for a single crystal. Below  ~100 K, the thermal conductivity is lower than that of any other crystalline solid to the best of our knowledge.

To identify the origin of this unusual behavior, we performed a number of additional characterizations on the BaTiS_3_ crystal. We first verified that the crystal is indeed an electrical insulator and possesses the expected stoichiometry; these properties were confirmed using electrical resistivity measurements and Rutherford backscattering (Supplementary Information). In particular, the Rutherford backscattering and refinements of the single-crystal XRD patterns indicate that the sulfur vacancy concentration cannot exceed a few percent. At these low vacancy concentrations, the scattering due to vacancies is well approximated by the Tamura formula^42^, and such a scattering rate cannot yield the observed temperature dependence and low values of thermal conductivity.

Next, we examined whether anharmonic damping could explain the observed trend using first-principles anharmonic phonon calculations based on the structure determined by single-crystal XRD. In this method, known as the temperature-dependent effective potential (TDEP) method, the renormalized harmonic force constants are determined at each temperature by fitting the force-displacement data sets obtained from density functional theory to a model Hamiltonian^43–45^. This method is capable of incorporating anharmonicity beyond the perturbative limits of conventional phonon calculations. As shown in Fig. 1f, this calculation matches the value of thermal conductivity at room temperature but predicts the opposite temperature trend of thermal conductivity. This result suggests that the anomalous thermal conductivity of BaTiS_3_ has a different origin.

### Local structure of BaTiS_3_

**Fig. 2:**
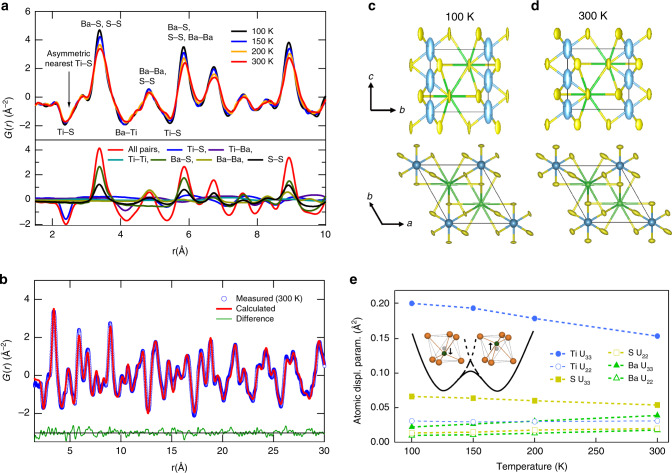
Pair distribution function (PDF) measured using neutron diffraction. **a** Measured PDF (*G*(*r*)) for several temperatures (top) and a breakdown of the pair distribution function (PDF) by atomic pairs for the 100 K refinement (bottom). The first few nearest neighbor distances are indicated. Since Ti has a negative neutron scattering length, the Ti-X peaks are negative. The nearest neighbor Ti-S peak is asymmetric and lacks temperature dependence. Note that other peaks that appear to lack temperature dependence actually consist of both positive and negative peaks as shown in the bottom panel, in contrast to the first Ti-S peak. **b** Measured PDF (blue circles) and best fit (red line) at 300 K. The residual difference between the measurements and fit is also shown (green line). Anisotropic distribution ellipsoids for 90 percent probability from the fit to PDF at **c** 100 K and **d** 300 K from different perspectives. The Ti distributions along the *c* axis (stretched blue ellipsoids) decrease in size with increasing temperature, the opposite of what is expected for thermal vibrations. **e** Derived atomic displacement parameters versus temperature. Note that the Ti-U_33_ parameter decreases with increasing temperature. The inset shows a schematic for the bimodal distribution of Ti atoms residing in shallow potential wells.

To quantitatively characterize the local structure of BaTiS_3_, we measured neutron powder diffraction patterns on the NOMAD instrument at the Spallation Neutron Source (see “Methods” section) and performed a pair distribution function (PDF) analysis using the PDFgui computer program^46^. Figure 2a shows the PDF (*G*(*r*)) for pair distances up to 10 Å. Significant peak anisotropy exists in the negative Ti-S peaks (note that because Ti has a negative neutron scattering length Ti-S and Ti-Ba peaks in the PDF appear negative). Typically, pair–pair correlation peaks decrease in intensity with increasing temperature because of thermal motion; this trend is observed for the Ba-S and S-S peaks but not for the Ti-S and Ti-Ba peaks. The absence of temperature dependence in these PDF peaks suggests a more complex site potential for the Ti atoms. Note that although other peaks such as the Ba-Ba and S-S peak near 4.84 Å also appear to show weak temperature dependence, this trend is due to the presence of other negative peaks. A breakdown of the PDF by atomic pairs determined from a refinement of the 100 K data is shown in the bottom panel of Fig. 2a (see Supplementary Information for details). Refinements of the PDFs were performed using both the *P*6_3_/*mmc* and *P*6_3_*mc*^37^ structures, but this choice of structure did not change the resulting local structure pair distributions.

  Figure 2b shows a representative fit to the PDF at 300 K using the *P*6_3_*mc* space group from 1.5 to 30 Å. The resulting anisotropic displacement parameters at 90% probability are illustrated in Fig. 2c, d. At 100 K (Fig. 2c) the anisotropic displacements of the Ti atoms are elongated along the *c* axis with a large (≫0.01 Å^2^) atomic displacement parameter Ti-U_33_ = 0.2(0.07) Å^2^, compared to Ti-U_22_ = 0.03(0.005) Å^2^, which is more consistent with typical thermal amplitudes. The S atoms are similarly elongated along the *c* axis with S-U_33_ = 0.07(0.01) Å^2^ compared to S-U_22_ = 0.014(0.002) Å^2^. On heating, however, these large U_33_ values decrease to 0.15(0.05) and 0.055(0.01) Å^2^ for Ti and S atoms, respectively, as shown in Fig. 2e. This behavior is opposite to that expected for thermal vibrations. The U_33_ and U_22_ parameters for the Ba atoms, on the other hand, are smaller and show the expected increase in heating from 100 to 300 K (Fig. 2e).

All of these results suggest the presence of increasingly large-amplitude displacements of one or both of the Ti and S atoms along the *c* axis as temperature decreases. We find that splitting the Ti position into two sites displaced by 0.15 Å along the *c* axis improves the fit at 100 K. A schematic illustrating such a bimodal distribution of the Ti atoms is shown in the inset of Fig. 2e. Given that the fits at higher temperatures are not improved by the bimodal model, it seems likely that above 100 K thermal vibrations in a soft potential are sufficient to explain the distributions. This observation may explain why the anharmonic phonon calculations agree well with experiments for 200 K and above (Fig. 1f) but qualitatively differ from the experiments at lower temperatures as the bimodal distribution develops.

**Fig. 3:**
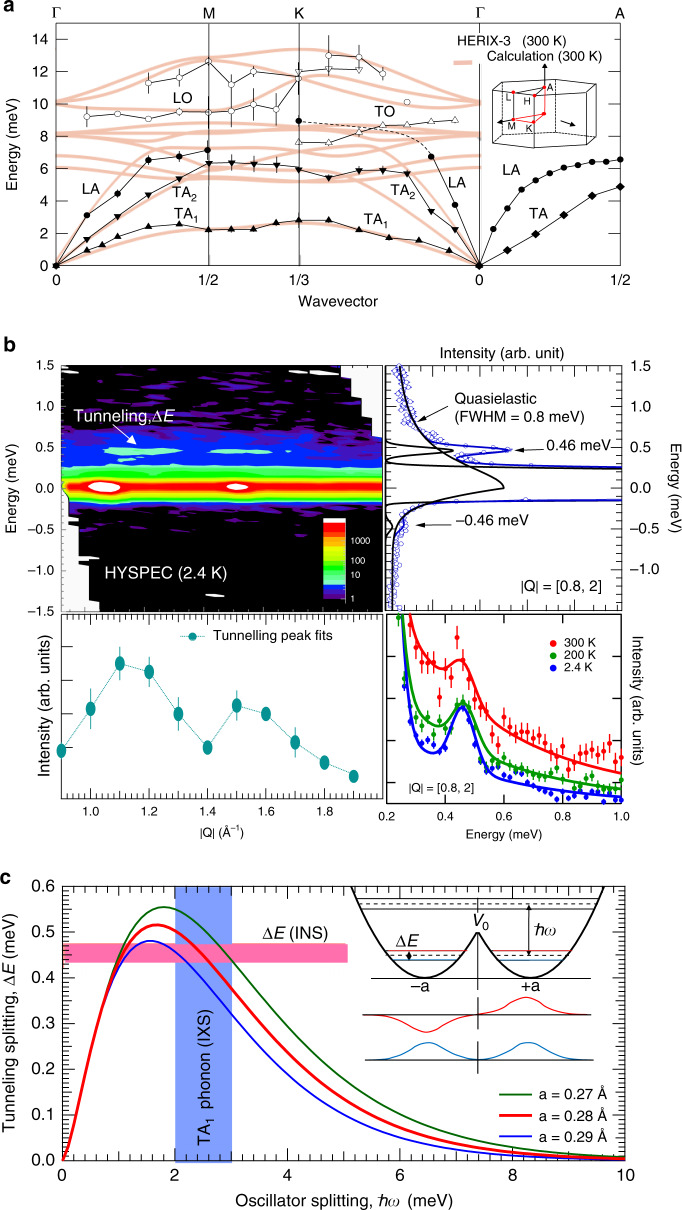
Lattice dynamics and atomic tunneling splitting. **a** Phonon dispersion measured using inelastic X-ray scattering for longitudinal (circles) and transverse (triangles) polarization, along with first-principles simulations (peach lines). The low-energy transverse acoustic phonon (TA_1_) is polarized along the *c* axis and is responsible for large-amplitude thermal displacements along this direction. **b** High-energy resolution inelastic neutron scattering spectra measured on BaTiS_3_ powder at 2.4 K. An excitation is observed at 0.46 meV. The top right panel shows the spectrum integrated over momentum, *Q*. The bottom left panel shows the tunneling peak intensity versus *Q*, and the bottom right panel shows the momentum-integrated spectrum at different temperatures. **c** Tunneling splitting energy, Δ*E*, versus oscillator energy (ℏ*ω*) from an analytical model for three double-well separation distances. The inset shows the double-well parameters and the symmetric and antisymmetric wave functions. The well separation predicted from the tunneling splitting (intersection of the red curve and pink horizontal bar) agrees with that inferred from the TA_1_ phonon energy (intersection of the red curve and blue vertical bar).

To assess the accuracy of our anharmonic phonon calculations and identify the origin of the dynamic displacements at 300 K, we measured the phonon dispersion of BaTiS_3_ using inelastic X-ray scattering on the HERIX-3 instrument at the Advanced Photon Source (see “Methods” section). As shown in Fig. 3a, the calculations agree with the measured dispersions (see Supplementary Information Fig. S10) with the exception of the LA and TA_2_ phonons along the *Γ* to K direction, in which case the measured dispersions are steeper than predicted. The largest displacements come from the low-energy (2 meV) TA_1_ phonon branch extending along *Γ*-M-K-*Γ*, which shows excellent agreement between theory and experiment. This phonon branch has large displacements polarized along the *c* axis and explains the large thermal displacements along this direction in Fig. 2e. However, the tendency for the Ti sites to develop bimodal distributions on cooling suggests that the Ti atoms are in shallow double-well potentials. On the other hand, static diffuse elastic scattering between the Bragg peaks in the IXS measurements was smaller by order 10^−8^ compared to the (200) Bragg intensity outside of the tails of this Bragg peak (see Supplementary Information Fig. S13). Hence, we can exclude any static diffuse scattering with high confidence, and the diffuse scattering observed by electron, X-ray, and neutron diffraction must be dynamic in origin.

### Phonons and an atomic tunneling system

Quantum mechanics predicts that a particle in double-well potential may tunnel through the potential barrier, leading to a tunneling splitting between the ground state and excited states^47^. A prominent example of atomic tunneling is the tunneling umbrella inversion of ammonia, which was used in the first MASER^48,49^. Translational tunneling of atoms other than hydrogen is rare in solids^34^ and has so far never been observed by scattering methods. Tunneling systems may scatter other phonons and have long been used to explain the thermal conductivity of solids at sub-1 K temperatures^29,50–53^. To obtain direct evidence of tunneling splitting, we performed high-energy resolution inelastic neutron scattering measurements on BaTiS_3_ powders using the HYSPEC instrument at the Spallation Neutron Source (see “Methods” section). As shown in Fig. 3b, a sharp low-energy excitation appears at 0.46 meV at a temperature of 2.4 K. That the intensity of the excitation peaks occurs above the Bragg peaks, the first of which is located at *Q* = 2*π*/*c* (*c* = *c* axis lattice parameter), indicates that it follows the crystal symmetry and that spatial correlations exist. The excitation energy remains constant to within error up to at least 200 K, as shown in the bottom right panel of Fig. 3b. This trend is consistent with atomic tunneling and rules out a thermally activated Arrhenius process.

We provide further evidence for the presence of the tunneling system by comparing the well separation distance inferred from the tunneling splitting energy and the TA_1_ phonon energy using a textbook model for tunneling in a double-harmonic well^47^. The tunneling splitting, *ΔE*, can be expressed in terms of the oscillator energy, *ℏω*, and the potential barrier, *V*_0_, as:

1 $$\Delta E=2\hslash \omega \sqrt{\frac{2{V}_{0}}{\hslash \omega \pi }}\exp \left(-\frac{2{V}_{0}}{\hslash \omega }\right).$$

For the double-harmonic well illustrated in Fig. 3c, the barrier potential can be written in terms of the oscillator frequency, *ω*, the atomic mass of Ti *m*, and separation distance *a*, using 2*V*_0_ = *mω*^2^*a*^2^. Figure 3c shows the expected tunneling splitting Δ*E* versus *ℏω* for several atomic separations. Since the low-energy TA_1_ phonon (Fig. 3a) contributes the large amplitudes to the Ti atomic motion for *T* > 100 K, we take its energy as representative of the oscillator splitting energy for the Ti atom, highlighted in the blue region in Fig. 3c. The 0.46 meV tunneling energy in red intersects this oscillator splitting at the curve corresponding to a separation distance of 0.28 Å. From the PDF analysis of Fig. 2, the mean square displacement parameter for Ti along the *c* axis in the double well at 100 K is U_33_ = 0.2 Å^2^ so that half the peak distance is $\sqrt{2{U}_{33}}/2=0.31$ Å. Therefore, the expected separation distance *a* is about half of the maximum extent of the thermal ellipsoid for Ti in Fig. 2c, a reasonable agreement given the simplicity of this model.

The Ti tunneling provides a natural explanation for the temperature dependence of the Ti-U_33_ displacement parameter. At sufficiently high temperatures, the atom is free to explore all positions allowed by a harmonic potential, which, by symmetry results in a distribution centered at the equilibrium position. As the temperature is decreased, when the occupation of the ground state and first excited tunneling state dominates, the central position is a local minimum for the wavefunction (Fig. 3c) and the maximum of probability ∣*ψ*^2^∣ to find the atom is split into both of the two off-center positions, which leads to an apparent large atomic displacement parameter (ADP) with respect to the average position (center of the well), even if the ADP within each well is minimal, in the zero-point motion limit. More generally, this behavior is a consequence of the dynamic instability encountered when a double-well potential develops^54,55^.

The origin of the unusual thermal conductivity trend can also be identified from this analysis. The calculation above yields an estimate of the potential barrier height as *V*_0_ = 2.3 meV, which is only about twice the ground state energy *ℏω*/2 of the oscillator. It is the low barrier potential arising from the softness of the TA_1_ phonon that results in a sufficiently high tunneling frequency to scatter thermal phonons transporting heat above cryogenic temperatures. Evidence of strong scattering of these thermal phonons can also be seen in the quasielastic scattering that appears just beneath the tunneling peak in Fig. 3b (broad inelastic intensity extending from elastic peak). This observation is consistent with the phonons being overdamped by resonant scattering with the tunneling states and naturally explains the low and glassy thermal conductivity.

Atomic two-level tunneling systems have been invoked to explain the low-temperature thermal properties of solids for over 40 years^29,52^. The model postulates that there exist atoms or groups of atoms that have available two nearly degenerate configurations between which they may tunnel^50,51^; thermal conductivity is then reduced because propagating vibrations are scattered by the two-level systems^53^. The low energy of the two-level systems in typical glasses confined their effects on thermal conductivity to sub-1 K temperatures. In contrast, our results show that sub-THz frequency atomic tunneling states can exist in high-quality single crystals, resulting in glass-like ultralow thermal conductivity well above cryogenic temperatures. The soft, symmetric double-well potential responsible for the high-frequency atomic tunneling provides a guide for identifying crystalline solids with intrinsically low thermal conductivity.

## Methods

### Growth of BaTiS_3_ single crystals

Single-crystal needles were grown using chemical vapor transport, similar to methods employed for other perovskite sulfides reported elsewhere^35,36^. The precursors, barium sulfide (Sigma-Aldrich, 99.9%), titanium (Alfa Aesar, 99.9%), and sulfur (Alfa Aesar, 99.999%) were mixed stoichiometrically and loaded in a quartz ampoule with the transporting agent iodine (Alfa Aesar 99.99%) in an argon-filled glove box. The tube was then evacuated and flame sealed using a blowtorch. The sealed ampoule was about 12 cm in length and 2 cm in diameter. The samples were heated to 1000 °C with a 0.3 °C/min ramp rate, held at 1000 °C for 60 h, and then quenched to room temperature using a sliding furnace setup with a cooling rate of 100 °C/min.

### Single-crystal XRD

The single-crystal X-ray diffraction was conducted on a Bruker SMART APEX DUO 3-circle platform diffractometer using monochromatic Mo K*α* radiation (*λ* = 0.71073 Å) with a fixed *χ* axis. For low-temperature measurements, APEX II CCD detector and Oxford Cryosystems Cryostream 700 apparatus were employed. A black needle-like specimen of BaTiS_3_ crystal, ~0.044 mm × 0.060 mm × 0.512 mm, was used as the sample for single-crystal XRD analysis. Paratone oil was used to help mount the sample in a Cryo-Loop. We scanned a complete hemisphere of points on omega (0.5°) with a 50 mm detector distance and 512  ×  512 pixels resolution. A total of 2520 frames were collected.

### *c-*axis identification

The *c*-axis direction of the needle-like crystal was confirmed by rotating the BaTiS_3_ crystal along an in-plane direction to identify the six-fold rotational axis of the crystal (see Supplementary Fig. S1). The XRD map with sample tilt and the rocking curve measurements were carried out in a Bruker D8 Advance X-ray diffractometer with parallel beam configuration and a Ge (440) 2-bounce monochromator for Cu K_*α*1_ radiation.

### Time-domain thermoreflectance

A thin Al layer (~40 nm) was deposited onto the BaTiS_3_ single crystal using electron-beam evaporation for TDTR measurements. TDTR splits an ultrafast laser pulse train into two beams, a pump and a probe beam. The pump beam is modulated at radio frequency and heats the sample. The surface temperature of the sample is monitored by the probe beam via the change of reflectance with temperature. The thermal conductivity of BaTiS_3_ is extracted by fitting a model to the measured signal, where the magnitude and phase depend on the thermal properties of each layer. Here we used a 1/*e*^2^ laser radius of 10 μm and a modulation frequency of 4.6 MHz. Both pump power and probe power were  maintained at 2.5 mW to limit the steady-state temperature rise to less than 20 K while still providing a signal to noise ratio of more than 50. More details can be found in the Supplementary Information.

### Ab initio calculations

We carried out first-principles simulations using the projector augmented wave (PAW) method^56^ as implemented in the Vienna Ab initio Simulation Package (VASP)^57–60^. Exchange-correlation was treated using the AM05 functional^61,62^, and the plane wave energy cutoff was set to 800 eV. We performed calculations on a temperature-volume grid consisting of 6 temperatures and 5 volumes sampled using 3 × 3 × 3 Monkhorst-Pack^63^ mesh of k-points. We employed a near-cubic non-diagonal supercell containing 240 atoms. We fully relaxed the structure for each volume until forces act on the atoms were less than 10^−7^ eV/Å. We calculated forces and displacements using a self-consistent procedure, consisting of four iterations, where an iteration consists of calculating normal modes, generating snapshots, calculating forces, and fitting force constants. For each iteration, a total of 250 snapshots were used to achieve  sufficient constraints to the IFCs and their convergence. Then, we minimized Helmholtz free energy *F*(*T*, *V*) at each temperature to find the equilibrium volume at each temperature. The phonon dispersions and thermal conductivity were calculated and analyzed with the temperature-dependent effective potential method (TDEP)^43–45^ by extracting second and third-order force constants.

### Neutron powder diffraction measurements

Approximately 200 mg of BaTiS_3_ powder was loaded in a 3-mm-diameter quartz capillary and sealed with a nut and ferrule lid. The capillary was loaded in the sample shifter carousel at the Nanoscale Order Materials Diffractometer (NOMAD) instrument^64^ at the Spallation Neutron Source. Data were collected at 100, 150, 200, and 300 K (controlled by an Argon cryostream) for a total of 4 h per temperature at 60 Hz setting. The NOMAD data reduction programs^64^ were used to normalize collected spectra against a vanadium rod, subtract background and container scattering signals, and produce data appropriate for Rietveld and pair distribution function (PDF) analyses. PDF data *G*(*r*) were produced by Sine Fourier transformation of the total scattering structure factor *S*(*Q*) data with the *Q* range of 0.2 to 31 Å^−1^. The resulting PDF was analyzed using the PDFgui computer program^46^.

### Inelastic X-ray scattering measurements

Phonon dispersion curves were measured using inelastic X-ray scattering (IXS) rather than neutron scattering because the crystal dimensions were too small for neutron scattering but well matched to IXS. Dispersion curves were measured from single crystals of BaTiS_3_ using the HERIX-3 X-ray spectrometer at the Advanced Photon Source (APS)^65–67^ with 21.657 keV (*λ* = 0.5725 Å) X-rays focused to a beam size of 20 μm. The energy resolution for the IXS scans was 2.1 meV FWHM. The X-ray attenuation length is about 190 μm for BaTiS_3_ with 21.6 keV X-rays, and we used crystals near this thickness (160 μm) to optimize the signal.

### Inelastic neutron scattering measurements

To determine the tunneling energy, high-energy-resolution measurements were performed on 10 g of BaTiS_3_ powder loaded in an aluminum sample that can use the HYSPEC time-of-flight cold neutron spectrometer at the Spallation Neutron Source of Oak Ridge National Laboratory^68^. Measurements were performed at *T* = 2, 100, 200, and 300 K with an incident neutron energy of *E*_i_ = 3.8 meV and an energy resolution of 0.06 meV (FWHM) at a neutron energy transfer of 0.46 meV, the energy of the tunneling splitting. Empty cans were also measured at each temperature and used to make background subtractions.

## Supplementary information

Supplementary Information

## Data Availability

The authors declare that the data supporting the findings of this study are available within the paper and its Supplementary Information files.
