# Supplementary material for: High frequency atomic tunneling yields ultralow and glass-like thermal conductivity in chalcogenide single crystals
Source: Nat Commun. 2020 Nov 27;11:6039. doi: 10.1038/s41467-020-19872-w (PMC7699621; doi:10.1038/s41467-020-19872-w)
Supplement: Supplementary file 1 — Supplementary Information [file 41467_2020_19872_MOESM1_ESM.pdf]

Note: This manuscript has been authored by UT-Battelle, LLC under Contract No. DE-AC05-00OR22725 with the U.S. Department of Energy. The United States Government retains and the publisher, by accepting the article for publication, acknowledges that the United States Government retains a non-exclusive, paid-up, irrevocable, world-wide license to publish or reproduce the published form of this manuscript, or allow others to do so, for United States Government purposes. The Department of Energy will provide public access to these results of federally sponsored research in accordance with the DOE Public Access Plan(http://energy.gov/downloads/doe-public-access-plan).

High frequency atomic tunneling yields ultralow and glass-like thermal conductivity in chalcogenide single crystals

Bo Sun,[1,2,*] Shanyuan Niu,[3,4,*] Raphael P. Hermann,[5,*] Jaeyun Moon,[1] Nina Shulumba,[1] Katharine Page,[6] Boyang Zhao,[3] Arashdeep S. Thind,[7] Krishnamurthy Mahalingam,[8] JoAnna Milam-Guerrero,[9] Ralf Haiges,[9,10] Matthew Mecklenburg,[11] Brent C. Melot,[9] Young-Dahl Jho,[12] Brandon M. Howe,[8] Rohan Mishra,[7,13] Ahmet Alatas,[14] Barry Winn,[6] Michael E. Manley,[5,†] Jayakanth Ravichandran,[3,15,†] and Austin J. Minnich[1,†]

[1]*Division of Engineering and Applied Science, California Institute of Technology, Pasadena, California 91125, USA*

[2]*Tsinghua-Berkeley Shenzhen Institute, Tsinghua University, Guangdong Provincial Key Laboratory of Thermal Management Engineering and Materials, Shenzhen, Guangdong 518055, China*

[3]*Mork Family Department of Chemical Engineering and Materials Science, University of Southern California, Los Angeles, California 90089, USA*

[4]*School of Earth, Energy and Environmental Sciences, Stanford University, Stanford, California 94305, USA*

[5]*Materials Science and Technology Division, Oak Ridge National Laboratory, Oak Ridge, Tennessee 37831, USA*

[6]*Neutron Scattering Division, Oak Ridge National Laboratory, Oak Ridge, Tennessee 37831, USA*

[7]*Institute of Materials Science and Engineering, Washington University in St. Louis, St. Louis, Missouri 63130, USA*

[8]*Materials and Manufacturing Directorate, Air Force Research Laboratory, Wright-Patterson AFB, Dayton, Ohio, USA*

[9]*Department of Chemistry, University of Southern California, Los Angeles, California 90089, USA*

[10]*Loker Hydrocarbon Research Institute, University of Southern California, Los Angeles, California 90089, USA*

[11]*Core Center of Excellence in Nano Imaging, University of Southern California, Los Angeles, California 90089, USA*

[12]*School of Electrical Engineering and Computer Science, Gwangju Institute of Science and Technology, Gwangju 61005, South Korea*

[13]*Department of Mechanical Engineering and Materials Science, Washington University in St. Louis, St. Louis, Missouri 63130, USA*

[14]*Advanced Photon Source, Argonne National Laboratory, Argonne, Illinois 60439, USA*

[15]*Ming Hsieh Department of Electrical Engineering, University of Southern California, Los Angeles, California 90089, USA*

[*] These authors contributed equally to this work

[†] e-mail: aminnich@caltech.edu; jayakanr@usc.edu; manleyme@ornl.gov

I. ADDITIONAL SAMPLE CHARACTERIZATION

A. Rotational x-ray diffraction (XRD) map

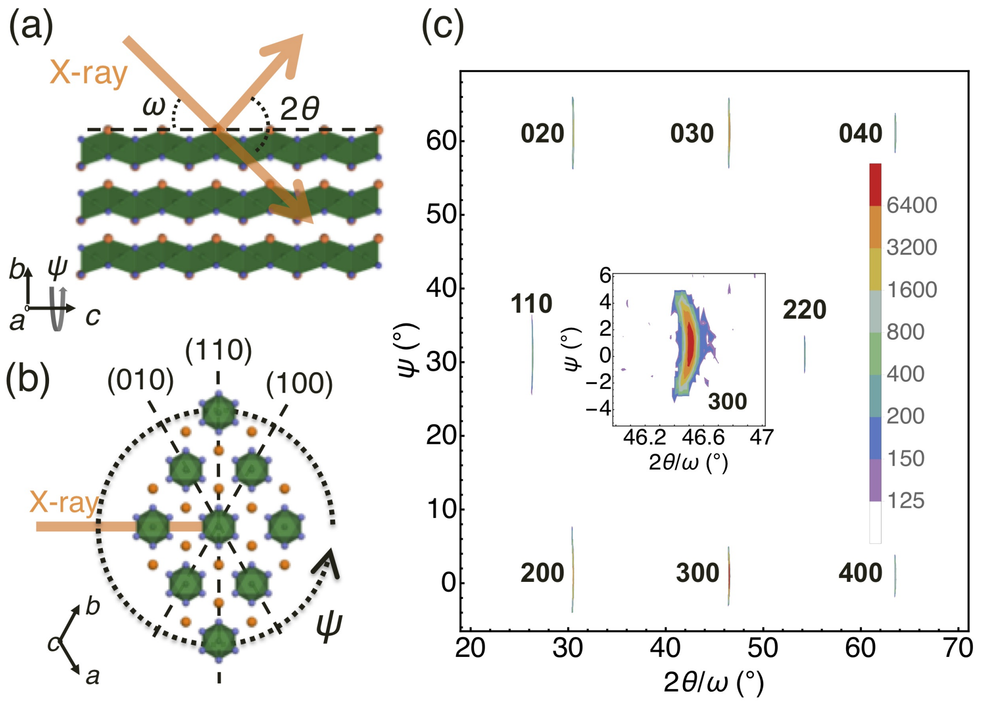

Fig. S1. Schematics for the configuration of the rotational XRD map projected along (a) a-axis and (b) c-axis. Orange and violet spheres represent Ba and S atoms, respectively. Octahedra formed by Ti and six surrounding S atoms are highlighted in green. (c) XRD map of a crystal to identify the crystallographic directions for the thermal conductivity measurements. ψ is the rotation angle of the crystal around the c axis. The colored scale bar indicates the intensity of XRD reflections in counts per second. The inset is the zoomed-in view of 300 reflection.

To identify the crystallographic directions of the crystal, we extended the method of out-of-plane Bragg scan in a thin film x-ray diffractometer to confirm the 6-fold rotation axis of the hexagonal structure. The rotational XRD map shown in Fig. S1(c) was constructed by a series of consecutive out-of-plane $2\theta/\omega$ scans taken as the crystal plate was rotating around the hypothesized c-axis. The illustrative schematic for the x-ray and crystal orientation projected along a- and c-axis are shown in Fig. S1(a) and (b), respectively. It is essentially a map of reciprocal space that is perpendicular to the rotating axis. The reciprocal lattice points are extended to long streaks along ψ due to the relatively poor divergence of the x-ray

beam in this direction in the instrument. All the reflections in Fig. S1(c) are $(hk0)$ type, and we can see a repetition of $\{100\}$ reflections when rotated 60 degrees away, confirming that the rotating axis is indeed the c-axis. We can also see $\{110\}$ reflections when the crystal is rotated 30 degrees away from the 100 facet. These observations agree well with the crystal symmetry. The absence of other reflections and the narrow dispersion of observed reflections indicate high crystalline quality.

B. Single crystal x-ray diffraction

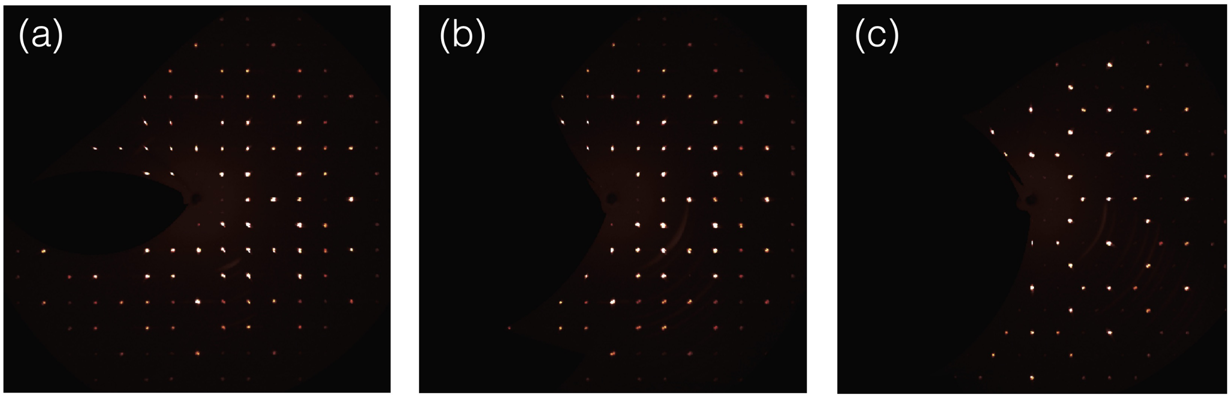

Fig. S2. Procession images from single crystal XRD measurement on a $BaTiS_3$ crystal along a (a), b (b) and c (c) axis projections.

C. Scanning transmission electron microscopy (STEM)

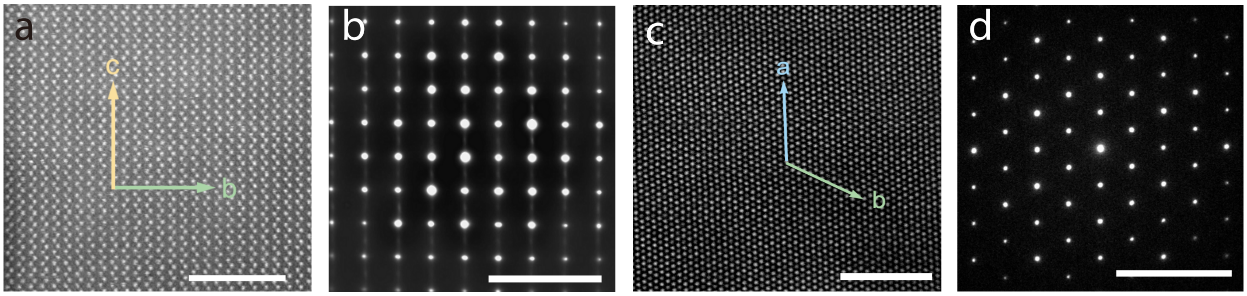

Fig. S3. (a) STEM image and (b) SAED pattern along the a axis of a $BaTiS_3$ crystal. (c) STEM image and (d) SAED pattern along the c axis. Scale bars: (a) and (c) 5 nm, (b) and (d) 5 nm^{-1}.

D. Electrical resistivity measurement

We measured the temperature dependence of electrical resistivity of a $BaTiS_3$ (BTS) crystal. Four contacts were made by directly painting silver epoxy onto the crystal surface, as shown in the inset picture in Fig. S4(a). Four-probe resistance was obtained by passing current through two end contacts and measuring voltage across the two inside contacts. Linear I-V curves were measured in the temperature range shown. The sample dimensions were measured in a microscope and electrical resistivity was calculated. The sample clearly shows semiconducting behavior with increased resistivity when cooled to lower temperatures, as shown in Fig. S4(a). The electronic contribution of thermal conductivity was estimated using the Wiedemann–Franz law. The largest electronic contribution to the total thermal conductivity measured by TDTR was less than 5% at all temperatures, as shown in Fig.S4(b). Hence, the electronic contribution to the thermal conductivity is negligible and the thermal transport is dominated by phonons.

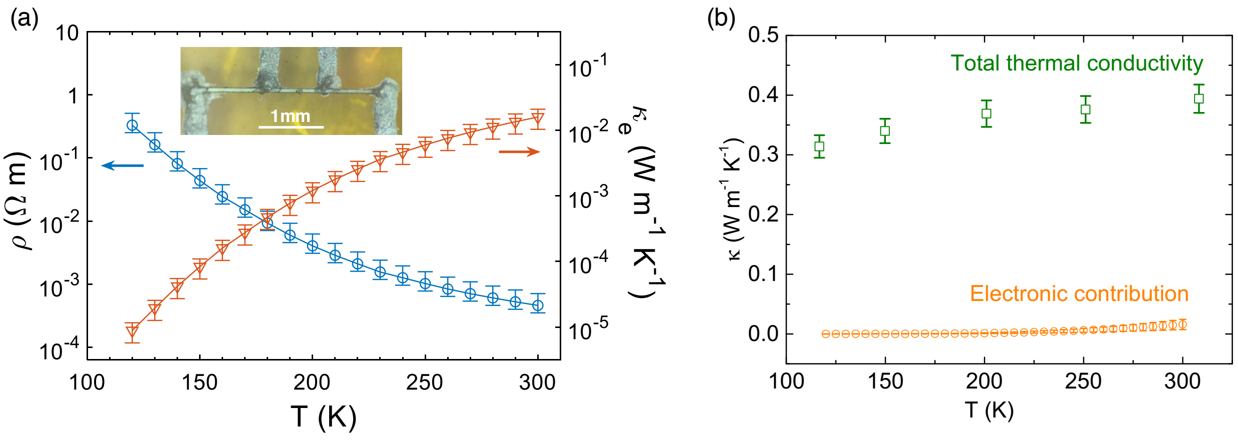

Fig. S4. (a) Measured electrical resistivity ρ and calculated electronic contribution of thermal conductivity κ_e of a $BaTiS_3$crystal as a function of temperature. The inset is an optical image of the sample used in the measurement. (b) Comparison of total thermal conductivity measured by TDTR with electronic contribution of thermal conductivity calculated from resistivity.

E. Rutherford Backscattering Spectrometry (RBS) for compositional analysis

We use RBS for quantitative determination of the composition of our $BaTiS_3$samples. In RBS analysis, samples are bombarded with He^{2+} ions at an energy in MeV range and the energy distribution and yield of the backscattered ions at a given angle are detected. The energies of backscattered ions depend both on the mass of atoms from which they are scattered as well as the the depth in the sample at which the collisions occur. The number of backscattered ions is directly proportional to the concentration of a given element. For $BaTiS_3$, we estimate that the uncertainty of RBS measurement can be as good as 2%. In Fig. S5, we show the RBS determination of the stoichiometry of $BaTiS_3$. We found that the nominal ratio (1:1:3) of $BaTiS_3$gives a good fit to our RBS measurements, and thus we determined that the composition of our sample is $BaTiS_3$ to within the uncertainity of the RBS measurement (2-3%).

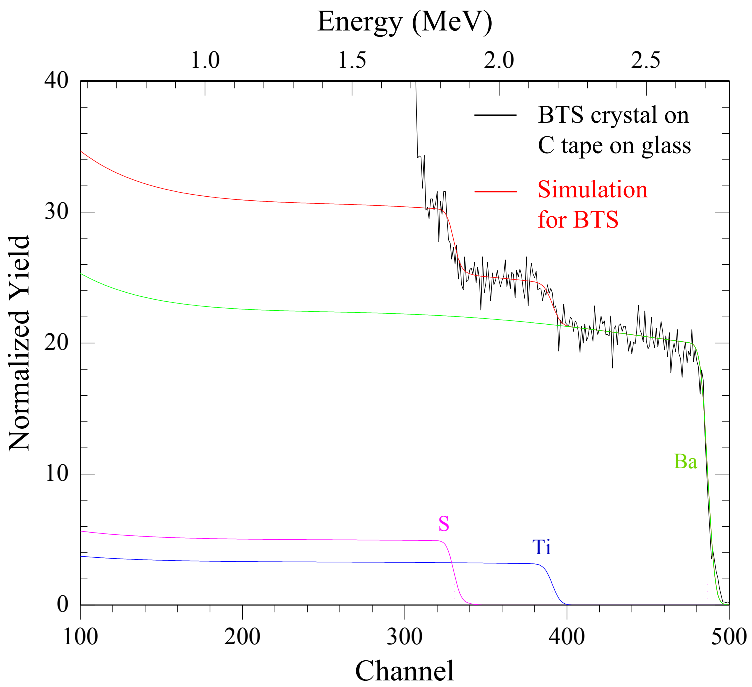

Fig. S5. RBS measurement on a $BaTiS_3$ platelet. Black line shows the RBS data. Purple, blue and green lines show the fitting of S, Ti and Ba elements, respectively. Red line is the simulated curve assuming that Ba:Ti:S is 1:1:3. The nominal stoichiometry of $BaTiS_3$shows a good fit.

II. TDTR MEASUREMENTS

A. Comparison of measured TDTR data of $BaTiS_3$ and glass

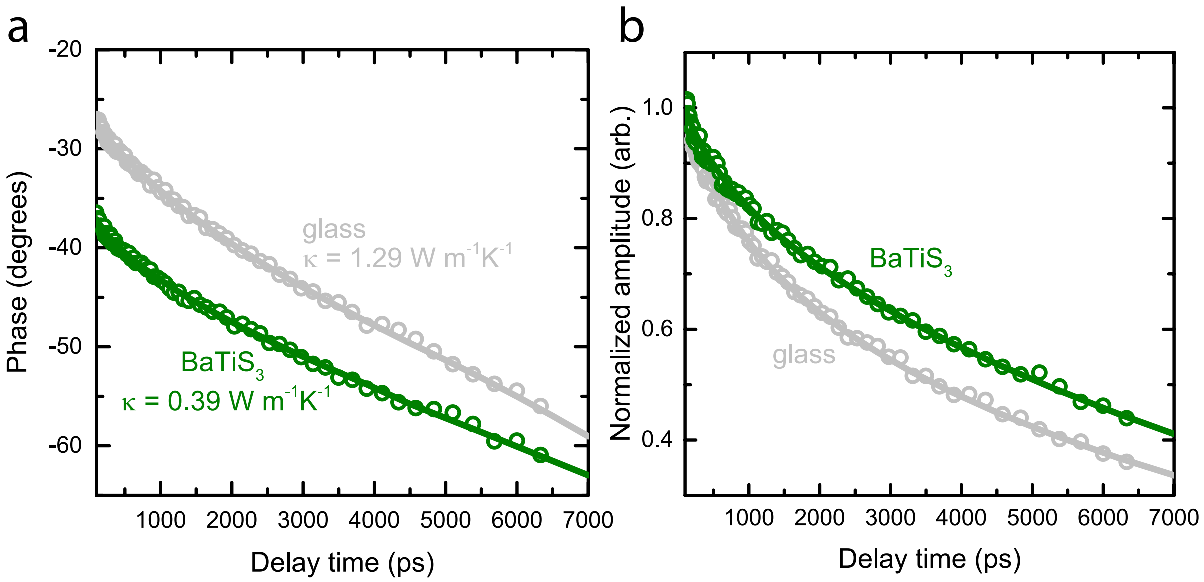

Fig. S6. (a) Phase and (b) amplitude versus delay time measured using TDTR of $BaTiS_3$ single crystal (green circles) and soda lime glass (grey circles) at room temperature along with the best fit from the thermal model (solid lines).

B. Steady state temperature rise

Steady state temperature rise (ΔT_{ss}) from pulse laser heating is a major concern when measuring bulk materials with low thermal conductivity using TDTR, which can easily reach tens or even hundreds of K depending on the thermal conductivity of substrate, laser beam spot size and absorbed laser power. As the fitting procedure in TDTR is highly sensitive to the temperature-dependent input thermal properties (volumetric heat capacity, thermal conductivity) of Al transducer and substrate, we normally limit ΔT_{ss} to less than 10 K, or 20 K at most for materials with ultralow thermal conductivity such as $BaTiS_3$. Temperature increases larger than this value can lead to inaccurate thermal conductivity measurement.

ΔT_{ss} can be estimated using: [1]

$$\Delta T_{ss} = \frac{P(1-R)}{2\sqrt{\pi} w_0 \kappa} \tag{1}$$

where P is incident laser power, R is the reflectance of sample surface, w_0 is $1/e^2$ radius of laser beam and κ is the thermal conductivity of substrate. This equation is an estimation for the case of quasi-1D heat flow where lateral heat spreading in Al transducer is not important. However, in our case, as $BaTiS_3$has ultralow thermal conductivity, lateral heat spreading in Al transducer is significant thus cannot be ignored.

We thus calculated ΔT_{ss} by setting the heating frequency to zero (no periodic component) in the thermal model for multilayer sample [1]. For a typical experiment, ΔT_{ss} is about half of that estimated using equation 1, indicating the effect of lateral heat spreading in Al transducer. The laser power we used was optimized as a compromise between signal to noise ratio and ΔT_{ss}. We used a total power of 4 mW (pump 2 mW and probe 2 mW), which gave us $\Delta T_{ss} < 20$ K while yielding a good signal to noise ratio of 50. The $1/e^2$ beam radii for pump and probe were 10 microns.

C. Heat capacity of $BaTiS_3$

The heat capacity of $BaTiS_3$ was determined using first-principles calculations as well as measured using PPMS. We show the results from both measured values and calculated values in Fig. S7. The two show a maximum deviation of 5%. In the uncertainty analysis, we put 5% as the uncertainty of heat capacity.

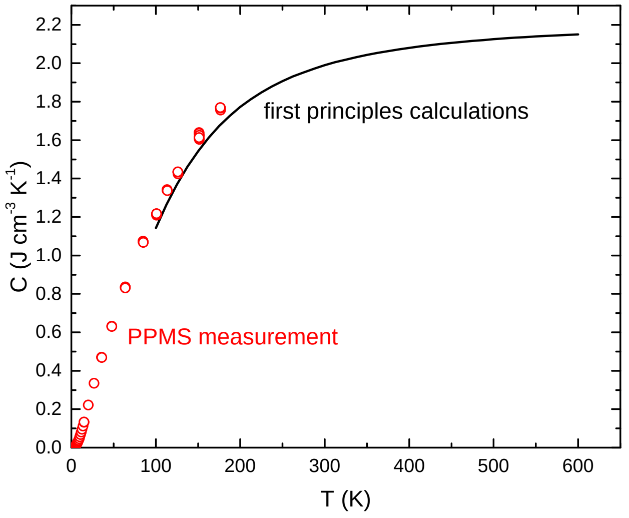

Fig. S7. Volumetric heat capacity of $BaTiS_3$ versus temperature measured using PPMS (red circles) and from first-principles calculations (line).

D. Sensitivity and Uncertainty Analysis

To evaluate the uncertainty of TDTR measurements, we first calculate the sensitivity of all parameters that are sensitive to TDTR measurements. The sensitivity parameter S_α is defined as:

$$S_\alpha = \frac{\partial \ln R}{\partial \ln \alpha} \tag{2}$$

where R is the absolute value of the ratio of in-phase and out-of-phase of the lock-in amplifier in TDTR measurements and α is the parameter in the thermal model, such as thermal conductivity, heat capacity, or thickness of each layer.

After all the sensitivity parameters are calculated, the measurement uncertainty of thermal conductivity can be calculated using the following equation:

$$\left(\frac{\Delta\kappa}{\kappa}\right)^2 = \sum\left(\frac{S_\alpha}{S_\kappa}\frac{\Delta\alpha}{\alpha}\right)^2 + \left(\frac{S_\phi}{S_\kappa}\delta\phi\right)^2 \tag{3}$$

where S_α is the sensitivity calculated using Eq.2 to parameters used in TDTR modeling, $\frac{\Delta\alpha}{\alpha}$ is the uncertainty for α, S_κ is the sensitivity to thermal conductivity of $BaTiS_3$, $\delta\phi$ is the phase uncertainty for lock-in detection,

From Fig. S8 the largest measurement uncertainties are those of the thickness and heat capacity of Al transducer and the heat capacity of $BaTiS_3$. We used 5% for the uncertainties of heat capacity of Al transducer and $BaTiS_3$, 3% for the uncertainties of thickness of Al

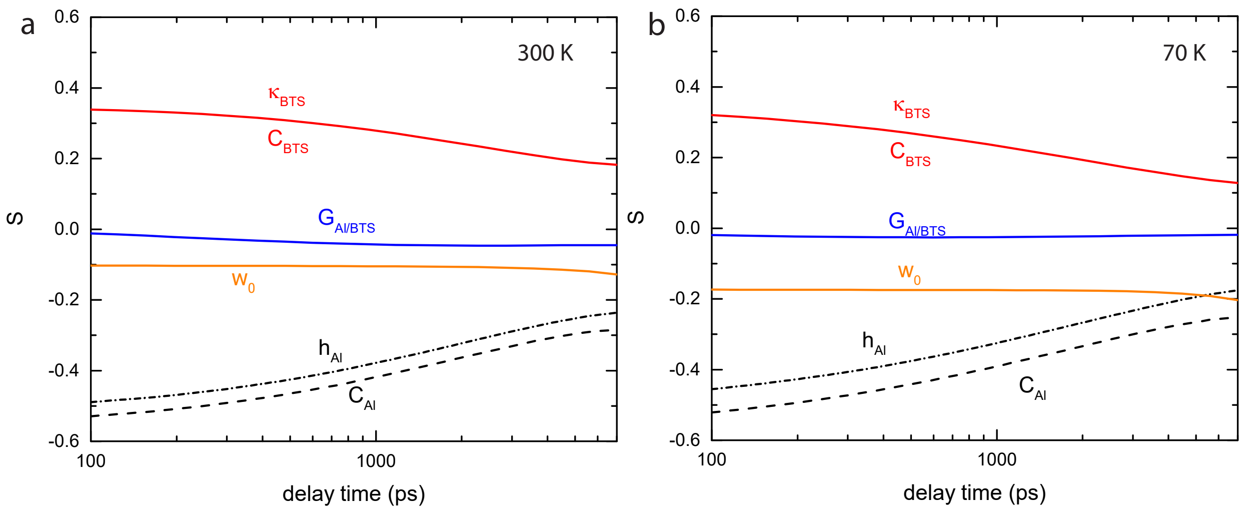

Fig. S8. TDTR measurement sensitivity to different parameters at room temperature (a) and at 70 K (b).

and 5% for the uncertainty for laser beam spot size. From Equation 3, TDTR measurement uncertainty for the thermal conductivity of $BaTiS_3$is about 6% at temperatures from 70 K to 400 K.

E. Transducer thickness

A thin Al transducer is preferred for the current parameters for TDTR measurements, as suggested by the sensitivity plot shown in Fig. S9. In our experiments, we use a thin Al transducer of thickness $\sim$ 40 nm. The thickness was determined by both picosecond acoustics and AFM.

In picosecond acoustics, the thickness of a film can be determined by measuring the roundtrip time of a thermoelastically generated stress pulse. An echo after the pulse was generated is observed after round trips through the Al film, and thus the Al transducer thickness can be calculated from time interval between echoes t_{echo} and the sound velocity of the film. From Fig. S10(a), we determined that thickness of Al film is 38.6 nm. We also employed AFM to verify the thickness of Al film. As shown in Fig. S10(b), Al thickness was measured to be 38.9 nm, which agrees the result from picosecond acoustics.

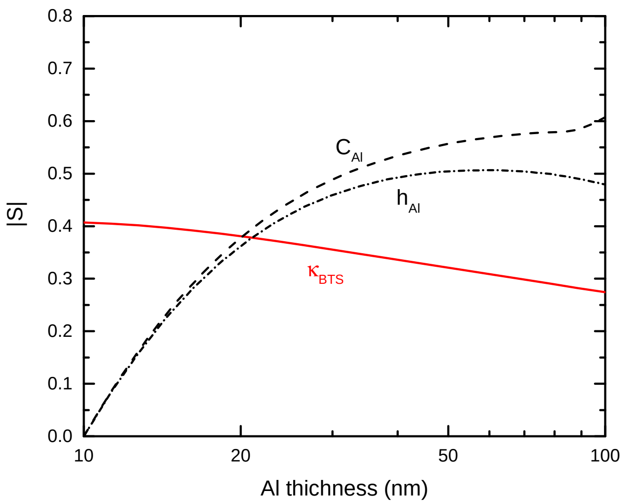

Fig. S9. TDTR measurement sensitivity to thermal conductivity of $BaTiS_3$, heat capacity and thickness of Al as a function of Al thickness

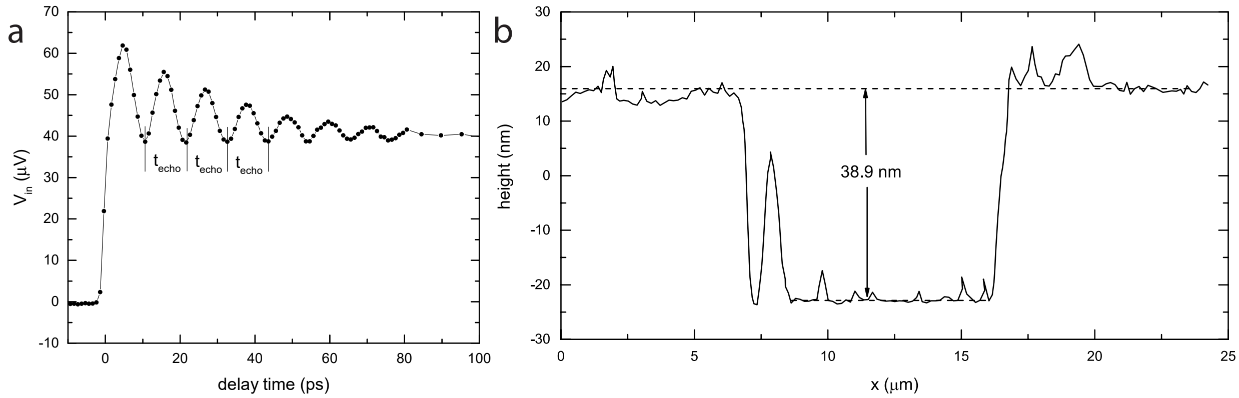

Fig. S10. (a) In-phase signal versus time on a Al/$BaTiS_3$ sample, showing the picoacoustic echoes used to determine film thickness. (b) AFM scan across a trench in Al transducer.

III. NEUTRON SCATTERING

The fit to the pair distribution function (PDF) calculated using PDFgui [2] follows a procedure very similar to a refinement of a diffraction pattern and involves starting with a model structure (already known from previous x-ray studies), instrument parameters, and then refining adjustable parameters including the lattice parameter and ADPs to obtain a best fit. The step by step procedure is described in the PDFgui user guide found here https://www.diffpy.org/doc/pdfgui/pdfgui.pdf. The fits were performed for the patterns over ranges of both 10 Å and 30 Å to ensure consistency, and larger range is used for the

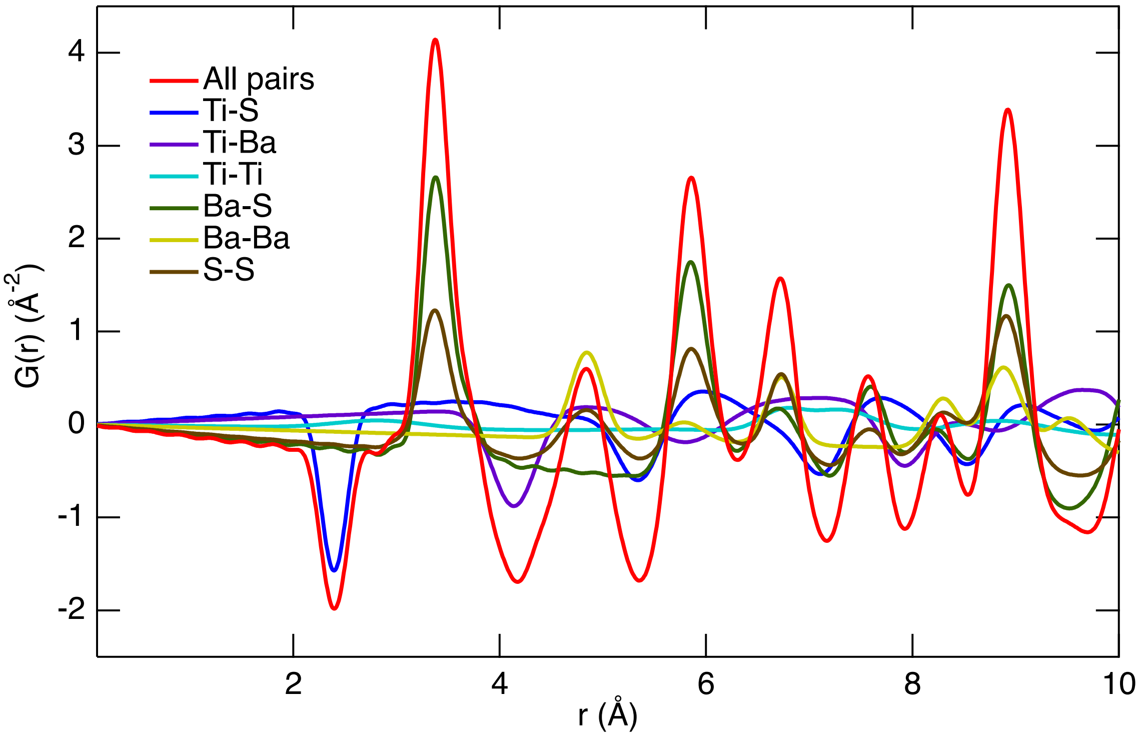

Fig. S11. Breakdown of the pair distribution function (PDF) by atomic pairs for the 100 K refinement of the $P6_3mc$ model of $BaTiS_3$.

final results. All results were consistent. Using the results of these fits the PDF can also be broken down into pair distribution functions for each atomic pair, as shown in Fig. S11. The partials allow the determination of which atomic pairs contribute to individual features in the measured PDF. However, with increasing pair distances the peaks exhibit more and more overlap making identification by direct inspection more challenging. Also, because Ti has a negative scattering strength it produces negative peaks in the PDF, which when positioned adjacent to positive peaks can produce the appearance of larger positive peaks than if no negative peaks were present. This situation occurs with the Ba-Ba, Ba-S peaks at $r = 4.84$ Å. The apparent height of this peak is partially due to the adjacent negative Ti-Ba and Ti-S peaks. Assuming a single site for the Ti atom the lowest R_w value (weighted profile residual) obtained was 0.13. Splitting the Ti atomic position into two sites along the c-axis lowered R_w further to 0.126. This amounts to changing the football shaped ellipsoids for Ti in Fig. 2 into more of an hourglass shape. This helps explain why the ADPs from single site fits appear to increase on cooling.

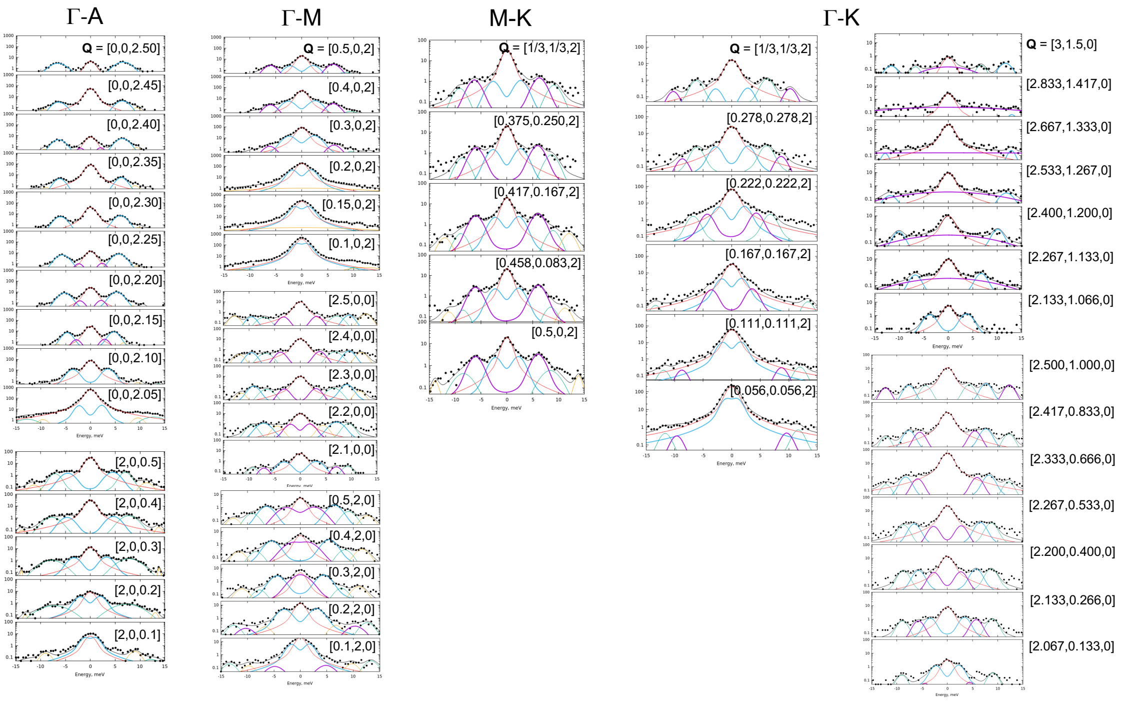

Fig. S12. Constant-Q scans measured using inelastic x-ray scattering on $BaTiS_3$. The scans were counted for 20 seconds per point. These fits were used to determine the phonon dispersion curves shown in Figure 3.

IV. INELASTIC X-RAY SCATTERING

Figure S12 shows the fits to all of the inelastic x-ray scattering data used to determine the phonon dispersion curves of $BaTiS_3$. Figure S13 shows elastic x-ray scattering in several regions of the Brillouin zone, demonstrating that diffuse elastic scattering that would arise from static disorder is not observed.

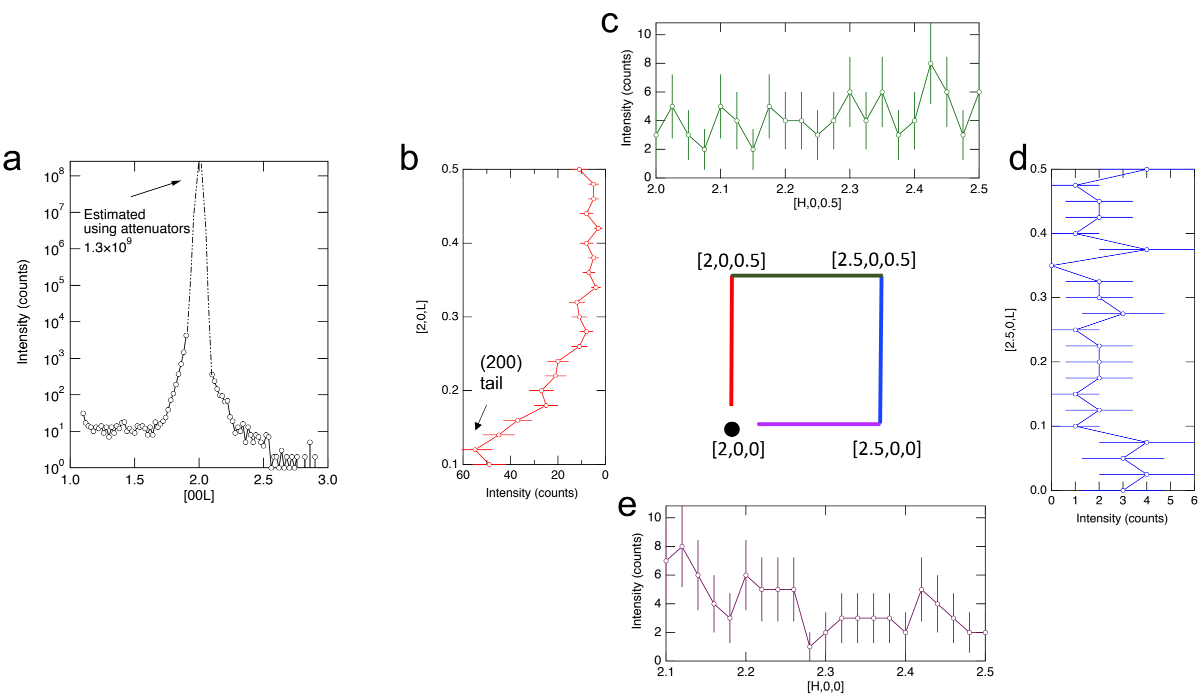

Fig. S13. Elastic ($E = 0$) scans measured using inelastic x-ray scattering on $BaTiS_3$. Each data point shown was counted for 5 seconds without attenuation. (a) Longitudinal scan through the (002) Bragg peak. The (002) Bragg peak could not be measured directly so attenuators were used to estimate the unattenuated intensity to be 1.3×10^9 counts. Similarly, the (200) Bragg peak (not shown) is estimated to have an unattenuated intensity of 4×10^8. (b)-(e) Elastic scans measured in a loop going from [2,0,0.1] to [2,0,0.5] to [2.5,0,0.5] to [2.5,0,0] to [2.1,0,0]. The signal away from the Bragg peaks is 8 to 9 orders of magnitude lower and registers only a few counts. If there were static displacement disorder there would be a strong diffuse elastic signal. Hence, this measurement excludes the presence of significant static disorder.

[1] D. G. Cahill, Review of Scientific Instruments **75**, 5119 (2004).

[2] C. L. Farrow, P. Juhas, J. W. Liu, D. Bryndin, E. S. Božin, J. Bloch, T. Proffen, and S. J. L. Billinge, Journal of Physics: Condensed Matter **19**, 335219.
